# Developmental and Evolutionary Lexicon Acquisition in Cognitive Agents/Robots with Grounding Principle: A Short Review

**DOI:** 10.1155/2016/8571265

**Published:** 2016-03-16

**Authors:** Nadia Rasheed, Shamsudin H. M. Amin

**Affiliations:** ^1^Faculty of Electrical Engineering, Universiti Teknologi Malaysia, 81310 Skudai, Johor, Malaysia; ^2^University College of Engineering and Technology, The Islamia University of Bahawalpur, Bahawalpur 63100, Pakistan; ^3^Centre for Artificial Intelligence & Robotics (CAIRO), Universiti Teknologi Malaysia, 54100 Kuala Lumpur, Malaysia

## Abstract

Grounded language acquisition is an important issue, particularly to facilitate human-robot interactions in an intelligent and effective way. The evolutionary and developmental language acquisition are two innovative and important methodologies for the grounding of language in cognitive agents or robots, the aim of which is to address current limitations in robot design. This paper concentrates on these two main modelling methods with the grounding principle for the acquisition of linguistic ability in cognitive agents or robots. This review not only presents a survey of the methodologies and relevant computational cognitive agents or robotic models, but also highlights the advantages and progress of these approaches for the language grounding issue.

## 1. Introduction

For the past two and a half decades, computational modelling for linguistic ability of agent or robot has focused on one of the important concerns for a symbol manipulation system to obtain the meaning of words, other than the dictionary style. This concern was pointed out by Harnad [1] as the symbol grounding problem. The adaptation of grounding methodology in agent or robotic language models then arose, as a reaction of dissatisfaction towards the pure symbolic approach. It is essentially an argument between researchers in favour of the grounded language approach that an association between the word and the physical experience provides the basics for linguistic communication [2]. Grounded language models have a symbol-physical world critical relation, while pure symbolic computation lacks this connection. As a consequence, with the grounded approach, the agent or robot has the ability to survive in real scenarios. This grounded approach of language modelling has changed the old-days concept of intelligent machines/robots as well as pushing robotic applications to the autonomous level.

In order to complement the theory validation necessity of the symbol grounding issue, many language models based on the grounded principle have been presented [3–12]. Some models used real robots, while others used simulated agents. In these grounded language models of cognitive agents or robots, different approaches and perspectives are considered and adopted. The linguistic ability of agents is built through physical interactions in all of the models.

If we look at the models from the late 1990s and the early 2000s, some of the early models were developed for scene description, in which phrases and sentences were generated through spatial language and visually grounded object description [11, 13–15]. In this line of research for visually grounded words (words with sensorimotor perception or concrete words), some models were inspired by children's learning methods. One of the earliest attempts for language grounding systems that was capable of directly processing recorded information from the environment is known as CELL (cross-channel early lexical learning) [11, 16]. This model was able to learn how to break speech into words and also capable of linking the words to visual shape and colour categories, which were acquired by listening to untranscribed infant-directed speech. However, this model was limited in terms of the number of objects in a visual input as it considered that only one object would be presented in a single scene. In contrast, children face a far more complex environment for learning. Another problem with this model was regarding referent understanding, as no tools were used to help in understanding the speaker referents. Yu et al. [17] worked on the same objective but with visual images of multiple objects. This model uses speakers' eye gaze to select specific regions in visual scenes.

As described earlier, different aspects have been considered for grounded acquisition of language in cognitive robots or agents. Some models focused on internal motivation and active exploratory behaviour to find developmental factors that favour language acquisition [18]. It has been shown that agents could develop their focus on vocal communication and language features through motivation towards the experience of situations [19].

In some models, the symbol grounding phenomenon was inspired by Pastra's two-way (top-down, bottom-up) grounding view [20–22]. According to this view, symbol grounding is a two-way grounding phenomenon. Firstly, the agent acquires a hierarchical composition of human behaviour through a bottom-up approach. Then, through a top-down approach (from symbol to sensorimotor representation), the agent gets the interpretation of symbolic structures.

This two-way grounding has been illustrated in an automatically built knowledge base, known as the PRAXICON, which consists of a semantic network of embodied concepts and pragmatic relations [23]. There are various representations for this knowledge base concept, such as linguistic, visual, motoric, and action centric network which are built from the findings of neuroscience [24]. Two-way grounding has also been implemented in the Ripley robot [25]. However, some important papers have addressed the issue of symbol grounding by considering a one-way or half-grounding mechanism, for example, one-way grounding of colour and shapes in the Toco robot [26].

Siskind [27] developed a model that grounds the meaning of verbs in perception by observing a visual scene through a fixed camera. The computational system would reconstruct the meaning after observing the scene, in which a human hand performs different actions on objects of different colours. This computer program is called LEONARD. This model used visually derived features to express the relationship between objects in the scene. This model has also used Talmy's force dynamic for relationships choice [28]. The semantics of basic verbs are modelled by temporal schemas that describe expected force dynamic interactions among objects. Some other models like Cannon and Cohen [29] also ground the meaning of verbs. Another model to learn actions is presented by Oladell and Huber [30]. In the presented approach, representational complexity is managed through symbolic feature representation generated by policies, affordance, and goals. This approach is implemented through the simulation of a robot arm and camera. The robot adds new policies, affordances, and goals to the dictionary structure.

Other than the factors and approaches described above, there are two very important methodologies that are adapted to ground symbol in cognitive agents or robots, which are the developmental and evolutionary acquisition of language. These two methodologies are not only important for the grounding of symbols but also useful for advancing robot controller design. In this paper, these two approaches to language grounding in cognitive agents/robots are explained in detail.

The paper is arranged as follows: in Section 2 the developmental language approach is discussed with a prototype model.  Section 3 explains the evolutionary methodology, such as developmental language acquisition methodology, and in this section an example model for this approach is also reviewed. The paper ends in Section 4 with concluding remarks.

## 2. Developmental Language Acquisition in Cognitive Agents/Robots

In this paradigm, cognitive agents or robots with higher-order behaviour emerge from the development and are layered on former ability. Linguistic ability is developed stepwise through direct interaction between the cognitive agents or robots and the social or physical world. The modelling approach for developmental language acquisition presented here is based on the connectionist computation as found in Cangelosi's models [3, 31–33]. This approach follows two basic mechanisms, in which the symbol grounding problem is solved in relevant models. Firstly, symbols (words) that are learned by the agent are linked to categorical representations. Moreover, these symbols also have logical associations with other symbols. Likewise, some symbols are grounded through direct interactions in perceptual, sensorimotor, social, and other internal states like emotions and motivations. Along with these directly grounded symbols, some symbols have a logical relation with a grounded symbol and are acquired just through language. This property provides productivity and generativity in language. For example, the two symbols, “TURN_FACE_RIGHT” and “TURN_FACE_LEFT”, which are directly grounded in the sensorimotor experience of an agent, generate a new word called “SHAKES_HEAD”. This new concept indirectly grounds in sensorimotor experience through the “symbol grounding transfer” mechanism (process) [1].

**Figure 1:**
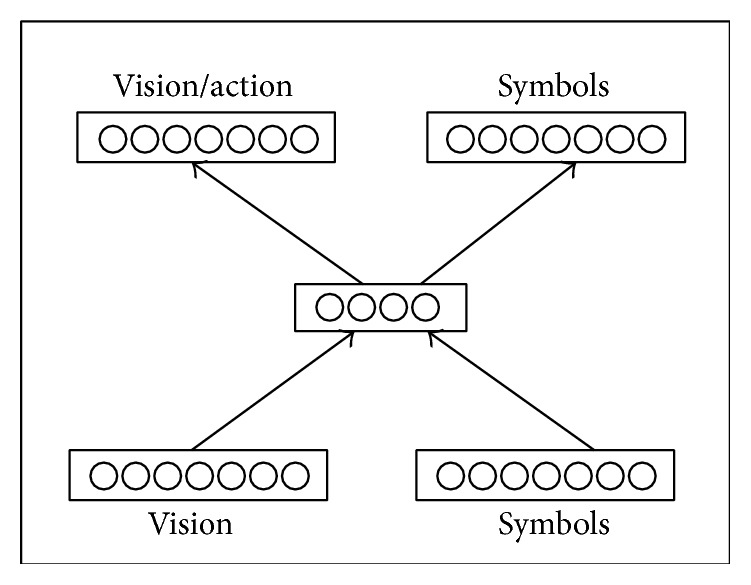
The dual-route neural network architecture [34].

The individuality of this grounded approach is that meanings of symbols emerge as a result of the imitation or sensorimotor interaction of agents that occurred for survival purpose. In the models of this approach, the agent's control architecture consists of an artificial neural network. Therefore, meaning has distributed representation, as with hidden unit activation patterns. The neural network models of this approach are designed in this manner, in which all capabilities (sensorimotor, cognitive, and linguistic) of agents are integrated in one architecture and language comprehension, and language production grounds in internal representations. A prototype of neural architecture used in these models is shown in Figure 1 [34]. These models typically have two types of inputs (vision/perceptual units and linguistic/speech units) and two output units (motion control units, linguistic utterances units), with at least one hidden layer. These models have different patterns of connection for hidden units and internal units, with different modular architectures.

With this design approach, syntactic and semantic structures emerge from the inputted stimuli for training and no explicit rules are used [35]. This approach adequately tackles the major issues recognized in the symbol grounding problem [36]. Other attributes of this approach are the ability to handle the word grounding phenomenon with effect of situatedness and embodiment in the language modelling system. It also provides bottom-up bootstrapping of behaviour in cognitive systems for meaning acquisition concern.

Although a number of models have been presented for acquisition of grounding language ability in agents or robots, there are only a few models presented in the developmental language acquisition research paradigm from the last decade until the present [32, 33, 37]. The progress of this modelling approach itself remains slow and most of the models only focused on lexicon acquisition belonging to verb category. Nevertheless, the adopted methodology in this line of models has been upgraded from the simulated agents to a real humanoid platform. In the next section, an important epigenetic robot computational model is reviewed, which has been presented for the grounding of words as a comprehensive view of this approach.

### 2.1. Epigenetic Robot Model with Grounding Transfer Method

**Figure 2:**
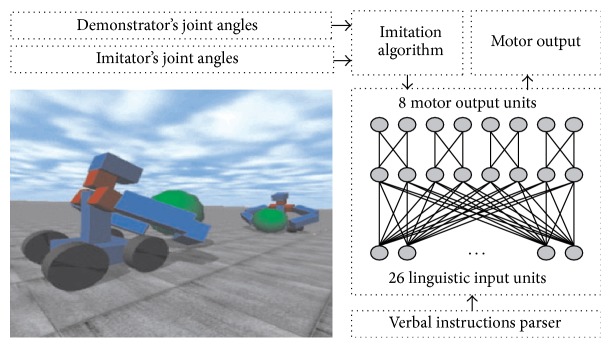
The bottom left picture shows the simulation environment. The diagram on the right shows the linguistic input from the parser to the neural controller and the corresponding motor output. The imitation algorithm makes a comparison between the demonstrator's joint angles and the imitator [37].

**Figure 3:**
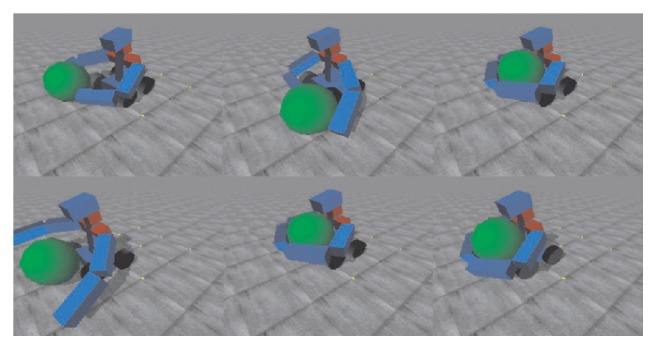
Demonstration of GRAB and CARRY words acquisition [37].

This robotic model was proposed by Cangelosi and Riga [37]. In this model, two teacher-learner robotic agents in a simulation scenario are embedded in the virtual environment. The simulation uses an open dynamics engine (ODE), which is an open source software library that enables the simulation of rigid body dynamics and joint types, with integrated collision detection with friction. The robot's body used in this model is loosely inspired by the humanoid robot with two three-segment arms (rotating shoulder, upper arm, and forearm), a torso, and four wheels, as shown in Figure 2 [37]. This simulation based robotic model could manipulate objects with two hands, as shown in Figure 3 [37].

In the simulation, the teacher agent firstly shows a basic action to the imitator (learner) agent. The teacher robot is manually programmed. The learner robotic agent is equipped with an artificial neural network controller through the direct grounding method and imitation grounds the actions. The learner neural controller with fully connected architecture consists of 26 input units, 8 output units, and one hidden layer which has 8 units. The details of the learner agents' functional modules, neural controller, and the three-dimensional view of robots and environment are shown in Figure 2 [37]. The imitation algorithm which is used for the learner robot is described as follows:

(1) ft+1=ft+gxt,yt

(2) gxt,yt=α21+exp⁡(−2βxt−yt−1α=scale,  β=gain.

The error back propagation algorithm is used in the training of the learner network. The simulation has three incremental stages, (1) basic action learning (BG), (2) higher-order grounding 1, and (3) higher-order grounding 2.

In the BG stage, the learner agent mimics the teacher's action. Names of actions are inputted to the learner's neural controller. The learner simultaneously learns the actions with names. This mechanism grounds basic action words. The actions or words like “CLOSE_RIGHT_ARM, OPEN_RIGHT_ARM, LIFT_LEFT_ARM, CLOSE_LEFT_ARM, LIFT_RIGHT_ARM, MOVE_FORWARD, MOVE_BACKWARD and OPEN_LEFT_ARM” are taught during the BG stage.

In higher-order grounding stages (HG1 and HG2), the learner receives the user's description of higher-order words and through the grounding transfer mechanism grounds inputted words indirectly, grounded in already acquired basic action words.

As an example, the word “GRAB” with the user's description, “GRAB [is] CLOSE_RIGHT_ARM, CLOSE_LEFT_ARM” is learned by following training cycles:

- First, the action word is inputted into the network and output is memorized.
- Previously memorized action word at output is mapped with higher-order word on input through training.
- In the next two cycles, steps 1 and 2 are repeated for the second action word.

The training details are 50 epochs for the BG stage, 100 epochs for HG1, and 150 epochs for HG2. In this study, the simulation results showed that the agent successfully learned the 6 basic actions and the 3 higher-order words and its knowledge level is enhanced by following quantitative language development stages.

## 3. Evolutionary Language Acquisition in Cognitive Agents/Robots

**Figure 4:**
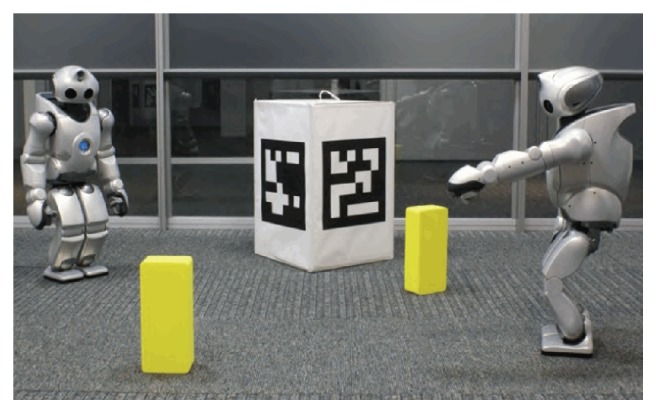
The language game scenario [44].

Shared communication systems provide a basis for modelling approaches of language evolution in robots. Communication through language games is widely used and becomes a successfully implemented modelling method for grounding the language in cognitive agents and robots. Language grounding through evaluation is mostly seen in Steels' robot game models [38–42]. A language game is a progression of verbal interactions between two agents situated in a specific context [38, 43], as shown in Figure 4 [44].

Language games help in solving a symbol grounding problem, as strong contexts constrain the meaning of words and allow robots to find the meaning easily. This approach offers the grounding of the meaning of symbols with contextual knowledge, as well as other aspects of language like embodiment nature and evaluation, through social interactions that could be implemented. Typically, in language games, the interaction between agents/robots is turn taking. Agents can play both roles, as a speaker and hearer. Grounding of symbols can be decomposed into three submethods: (1) sensing and preprocessing of data, (2) categorization of perceptual data, and (3) tagging. Similarly, in the language models, agents perceive the context of objects or actions through their sensors, extract feature vectors, and categorize their meaning. Later, a lexicon is created through topic selection.

The sensorimotor stimulation in games could be based on a scene acquired by a camera, with activation of an infrared, sonar, or simple light sensor. Usually, the raw data is processed, in which the region of interest is extracted through an algorithm, which then creates the feature vectors.

**Figure 5:**
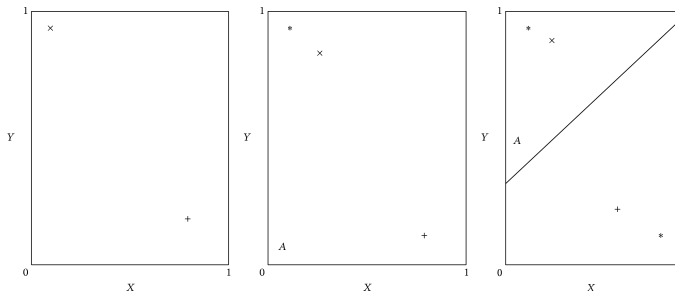
This figure shows an illustration of three successive discrimination games by a prototype representation. Three cases of a combined feature space and a conceptual space are shown [43].

In categorizing the preprocessed perceptual data in agents or robots, different techniques are used, such as the neural network and discrimination game. Discrimination games are one of the most frequently used methods for the categorization of perceptual data [43, 45]. The discrimination game categorizes the sensorimotor experience in the way that the category differentiates the experiences. An illustration of this game is shown in Figure 5 [43]. If a distinctive category is found during the perceptual experiences, the game is a success. If the game fails, a new category is formed. In this way, agents stretch perceptual category.

To summarize, a language game can be defined as follows. After perceiving the context and extracting the feature vector by speaker and hearer, robots would categorize the meaning by using a discrimination game. In the categorization step, the speaker selects one object as the topic of conversation and transforms its conceptualization into an utterance. The hearer must parse the utterance, then reconstruct its meaning, and map it into the hearer's own perceptual experience of the world. Games may fail in cases where diagnostics and repair strategies are used by the speaker and hearer to expand lexicon. Thus, by adjusting and aligning their language systems, they may be more successful in the future.

Discrimination games were originally implemented using a binary tree [46]. Other representations like radial basis function networks and neural networks were also applied in this game for perception categorization of grounded language models [41, 47].

One of the important language games used in experiments with robots is a guessing game. In a guessing game, the speaker robot points to an object, while the hearer robot draws attention to the indicated object in the environment. The hearer robot perceives and conceptualizes the situation and tries to guess the speaker's desires. An evaluation of the hearer's guess is made by the speaker robot and corrective feedback is provided in case of a mismatch.

Along with the above-described language games, there are also other games, such as the classification game, motion game, naming game, and observational game which have all been implemented in robotic models from a language grounding perspective [38, 48]. Evolutionary game models push the state of the art in the robotics field and other relevant fields; however, progress still remains steady. The earliest models employed a simple mobile robot, while the latest models use a humanoid platform. Another boost for this research domain comes from the emergence of grammatical structure in language game models which was restricted to just words grounding in earlier models. The latest language games experiments have employed a humanoid platform, and also grammar plays a role in these models to satisfy the language composition property [49–54].

In the next section, an important robotic experiment is discussed, which implements the language evaluation phenomenon in cognitive robots with the grounding principle for action category of words and provides a prototype example for the above-described language game methodology.

### 3.1. Action Game (Sony QRIO Humanoid Robot)

The game experiment which is presented here is called action game. In action game, which is a situated embodied game, humanoid robots ask each other to perform different bodily actions. In the experiment, they used Sony QRIO humanoid robots [55] to have a clear understanding of the operation, function, and nature of body image [49]. In the experiment, the robots played action games through diagnostic and corrective strategies. They progressively organized a lexicon, even when they did not have any prior list of names and categories of visually controlled body movements of others and never knew the relation among visual images of motor behaviours. In the experiment, bidirectional mapping between the motor and visual domain was also carried out.

**Figure 6:**
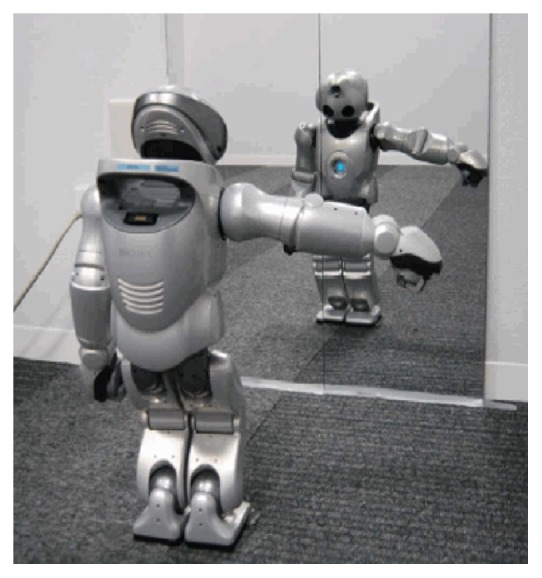
In the experiment the humanoid robot stands in front of a mirror and observes the visual body images of different actions [49].

**Figure 7:**
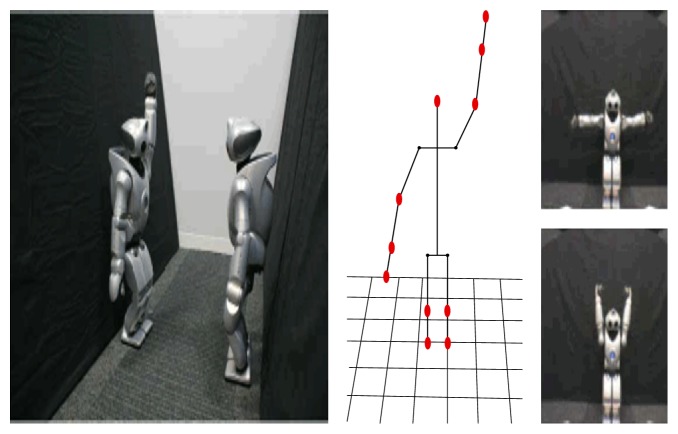
The action game experimental setup is shown. The central image shows the expected motor body image. The right one shows the postures that were seen through camera of another robot [49].

Specifically, in the beginning, the robot stood in front of the mirror and acquired the relation between its body image and mirror-based own visual body image, as shown in Figure 6 [49]. After the acquisition of the relation between body images, the robots engaged in the game by asking each other to take certain positions, and then feedback was given, to state whether this was achieved or not. Relevant experimental steps are shown in Figure 7 [49].

Since all the robots have the same body structures, they can categorize the body image of the other robots, from the reversal of perspective. In this game, the agents establish and adapt the network that links body representation, action, perception, and language. In this experiment, coordination between agents' visual and motor behaviour is achieved through language and observation in the mirror. This experiment has shown that any type of concept acquisition is possible through a language game even if it is an action word or context property.

## 4. Conclusions

In this paper, two main approaches of symbol grounding, developmental and evolutionary language acquisition, in cognitive agents or robots are reviewed. The developmental language method adopts a stepwise enhancement of linguistic ability, whereas the evolutionary game models emphasize the acquisition of language, with joint social interactions between agents or robots. It is proven from the survey that both of these approaches provide a solution to the issues relevant to the symbol grounding problem, while advancing robotic communication systems.

However, progress continues in both paradigms but at a sluggish speed particularly in the developmental language area. In particular, developmental language models were mainly focused on lexicon acquisition (basic and higher categories) in cognitive agents/robots, whereas in language game models progress could be seen from word acquisition to emergence of grammatical structures. On the contrary, methodological advancement for the robotic platform (from mobile robot to humanoid, simulated agents to humanoid robot) can be seen in relevant models in both paradigms, respectively.

Indeed, language is a very complex and broad phenomenon; therefore there remains much to work out for the grounding of language in cognitive agents/robotic systems with these approaches.
